# Impulsive suicide in methamphetamine intoxication and self-reported ADHD: a case report

**DOI:** 10.1007/s00115-025-01882-y

**Published:** 2025-08-05

**Authors:** Max Schallenberg, Jörg Pietsch, Maik Spreer, Maximilian Pilhatsch, Johannes Petzold

**Affiliations:** 1 Department of Psychiatry and Psychotherapy, Carl Gustav Carus University Hospital Dresden, Carl Gustav Carus Faculty of Medicine Dresden, Dresden University of Technology, Fetscherstr. 74, 01307 Dresden, Germany; 2 Department of Forensic Medicine, Carl Gustav Carus University Hospital Dresden, Carl Gustav Carus Faculty of Medicine Dresden, Dresden University of Technology, Dresden, Germany; 3 Department of Psychiatry and Psychotherapy, Städtisches Klinikum Dresden, Dresden, Germany

## Anamnesis

The patient’s history, summarized in Table 1, includes a challenging childhood marked by familial discord and attention-deficit hyperactivity disorder (ADHD), which was diagnosed via psychiatric evaluations but was never treated. Initial exposure to addictive substances began with alcohol consumption, escalating in early adolescence to daily use of alcohol (2–3 beers), tobacco (20 cigarettes), cannabis (1–2 cigarettes), and methamphetamine (1–2 lines), as well as biweekly oral use of methylenedioxymethamphetamine (MDMA). After completing school and during training, this consumption pattern continued and escalated to include occasional use of cocaine, heroin, and lysergic acid diethylamide (LSD).

**Table 1:** Patient timeline

Event	Age in years	Details
ADHD diagnosis	6–8	Via psychiatric evaluations, no re-evaluation in adulthood, never treated
Onset of daily substance use	12–15	Alcohol, tobacco, cannabis, methamphetamine, MDMA
First inpatient detoxification	15	For psychotic symptoms, polysubstance use continued after discharge
Suicide attempt	19	During substance-induced psychosis, required surgical intervention, followed by polysubstance use, unstable employment, relationship changes, criminal offences, periods of homelessness
Impulsive suicide	28	During methamphetamine intoxication

Shortly after completing the first inpatient detoxification, initiated due to psychotic symptoms such as hallucinations and delusions, the patient resumed consumption of psychostimulants and reported using substances for relaxation, mood regulation, and performance enhancement. The patient exhibited impulsivity and restlessness during multiple inpatient treatments, where methamphetamine was detected in urine ranging from 0.8 to 1.9 µg/mL. Impulsivity and restlessness were also documented during sustained abstinence at the end of a 4-week inpatient withdrawal treatment. Because the patient repeatedly disengaged from therapy, ADHD was not re-evaluated in adulthood, preventing diagnostic clarity given the overlap between methamphetamine-related symptoms and ADHD phenomenology.

The patient’s social situation in adulthood was characterized by unstable employment, relationship changes, criminal offenses, and periods of homelessness. There was one known suicide attempt during a substance-induced psychotic episode, resulting in injuries that necessitated surgical treatment. Otherwise, the patient’s medical history was unremarkable.

## Clinical course

The patient presented to a healthcare facility with somatic discomfort following excessive methamphetamine consumption. Initially cooperative, the patient rapidly deteriorated, displaying disorganized behavior, verbal aggression, and paranoid ideation, consistent with methamphetamine-induced intoxication. Despite multiple verbal de-escalation attempts, including offers of medication, the patient’s agitation continued to escalate. The patient was observed pacing and attempting to access restricted areas, where the patient seized a dangerous object and threatened staff. The patient suddenly opened an unlocked window, jumped out, and succumbed to traumatic injuries despite resuscitative efforts. Forensic toxicology with high-performance liquid chromatography/mass spectrometry quantified a blood methamphetamine concentration of 1.51 µg/mL. Except for amphetamine (0.089 µg/mL), no other substances (cocaine, cannabis, opioids, benzodiazepines, barbiturates, and alcohol) were detected.

## Discussion

### Methamphetamine use disorder or ADHD

This case report illustrates the tragic convergence of possible childhood ADHD, chronic methamphetamine use, and an impulsive suicide, building on the well-established associations between methamphetamine use and increased suicide risk [9, 10, 14, 19, 24] as well as the higher prevalence of ADHD [7, 21, 22]. The person-centered description intends to raise awareness among healthcare providers of how these associations may culminate in an impulsive suicidal act, defined as a rapid, unplanned action to kill oneself, distinct from excited delirium or substance-induced psychosis [37].

However, the available data from this forensic postmortem study do not enable a definitive conclusion to be made on whether ADHD contributed to the patient’s neuropsychiatric problems. First, ADHD diagnosed by a private psychiatrist in childhood was self-reported by the patient in adulthood; moreover, information on whether structured interviews or ADHD-specific questionnaires were conducted was lacking. Second, diagnosing ADHD in the context of methamphetamine use is highly challenging due to their substantial phenomenological overlap, including inattention, hyperactivity, impulsivity, and emotional instability [21, 22]. A reliable ADHD diagnosis requires an abstinence period of several months to help distinguish between ADHD and substance-induced symptoms [21, 22]. Without such abstinence, the validity and severity of the patient’s ADHD diagnosis remain questionable, particularly given the risk of overdiagnosis by clinicians who may overlook the complexities of differential diagnosis in substance-using populations [21, 29].

The missing re-evaluation of childhood ADHD in adulthood may also reflect systemic barriers, including stigma surrounding ADHD diagnosis and treatment, which may deter patients and families from seeking or continuing care [35]. Service fragmentation, such as poor coordination between child and adult mental health services or between addiction and psychiatric care, further limits access to integrated care. For example, regional disparities in access to specialized dual-diagnosis programs often result in patients receiving fragmented care, with substance use treatment prioritized over ADHD management [18, 29]. These barriers likely contributed to the diagnostic uncertainty regarding the patient’s ADHD, which may have promoted substance use, ultimately worsening psychosocial and clinical outcomes [12].

### Stimulant treatment for ADHD

Patients with ADHD have a markedly increased risk of developing a substance dependence, particularly to psychostimulants, partly driven by self-medication attempts [7, 21, 22]. Low-dose methamphetamine, unlike high-dose use, may have therapeutic effects on ADHD, as evidenced by its authorization for ADHD treatment in the United States [17]. Appropriate ADHD treatment during adolescence or adulthood is associated with lower risks of current and future substance-related problems [32]. At the same time, new research demonstrates that one quarter of adults who are prescribed stimulants, especially those being prescribed amphetamines, may misuse them, with 1 in 10 possibly having prescription stimulant use disorder [16].

The risk of misuse is also relevant to this case, as the patient engaged in polysubstance use, a common practice among people with methamphetamine use disorder [3, 15]. Individuals may use multiple substances to enhance their desired effects or counteract undesired effects, such as withdrawal symptoms [3, 15]. Therefore, the reasons for combining certain substances and the presence of other substance use disorders should be explored to provide integrated treatments for substance use disorders and related psychiatric conditions, such as ADHD. Inadequate management of either condition exacerbates mental instability, undermines therapeutic progress, impairs overall recovery, and elevates the risk of relapse [31, 38, 40]. Unsurprisingly, studies indicate that relapse rates in methamphetamine dependence exceed 60% within 1 year of receiving withdrawal treatment without psychosocial interventions [4, 27].

### Treatment of methamphetamine use disorder and ADHD

Despite a guideline recommendation of using pharmacotherapy, including stimulant medication, for ADHD in individuals with comorbid stimulant use disorder [38], the use of stimulant medication remains controversial due to limited clinical data supporting its efficacy and safety, particularly in adolescence [5, 20]. Yet, the lack of ADHD-specific pharmacotherapy, such as methylphenidate or atomoxetine, could exacerbate cognitive impairments and heighten suicide risk in patients with a dual diagnosis of ADHD and methamphetamine use disorder [23, 30, 33, 34].

Research suggests that non-pharmacological interventions tailored for substance use disorder and ADHD, such as motivational interviewing, contingency management, and cognitive behavioral therapy, may improve treatment adherence and reduce substance use [38, 40]. These interventions aim to address impulsivity and executive dysfunction through structured skill-building and psychosocial support. Ongoing clinical trials, such as the International Naturalistic Cohort Study of ADHD and SUD (INCAS), are investigating integrated treatment models to establish evidence-based frameworks for managing this dual diagnosis [5].

### Neurobiology of methamphetamine-related suicide

The patient’s blood methamphetamine concentration of 1.51 µg/mL at the time of the impulsive suicide was above the lethal threshold (> 1 µg/mL) as per toxicological guidelines [25, 36]. This aligns with the literature describing neuroadaptive dopaminergic changes in chronic users, potentially conferring paradoxical tolerance to toxic effects while exacerbating neuropsychiatric risk [34, 36]. For instance, the risk of psychosis is approximately 11 times higher in methamphetamine users than in the general population [26]. The patient’s suicide was likely driven by methamphetamine-induced psychosis and heightened impulsivity associated with methamphetamine use, which are known to precipitate suicidal behavior [9, 13, 24].

Neurobiologically, acute methamphetamine use induces rapid dopamine and noradrenaline surges that overstimulate the limbic system and disrupt prefrontal executive functions, possibly resulting in agitation, impulsivity, and psychotic symptoms [2, 3, 15]. By contrast, chronic methamphetamine use leads to neuroadaptations, such as downregulation of dopamine receptors and reduced prefrontal cortical activity, promoting impulsivity and poor decision-making over time [34, 39]. Both acute and chronic methamphetamine use contribute to behavioral dysregulation but differ in their temporal dynamics and therapeutic implications: Acute toxicity requires immediate stabilization, while chronic changes necessitate long-term neurorehabilitation [3, 34]. Methamphetamine-induced hypofrontal dysfunction can exacerbate pre-existing psychiatric conditions, particularly psychotic symptoms and affective disinhibition [39]. A substantial body of literature documents an elevated risk of suicide among methamphetamine users, particularly among those who have previously attempted suicide [8, 9, 11, 24], with contributing factors being impulsivity, psychotic symptoms, and affective dysregulation [9, 11, 13, 19].

### Acute management of methamphetamine intoxication

Methamphetamine intoxication, potentially accompanied by other substance use, is a medical emergency that can present with sympathomimetic toxicity (e.g., hyperthermia and rhabdomyolysis with subsequent kidney failure and hyperkalemia; cardiac arrhythmias, myocardial infarction, stroke, seizures) and psychological symptoms (e.g., hypervigilance, panic attacks, psychotic symptoms, violent outbursts; [3, 15]). The patient, who presented with somatic discomfort, was initially cooperative but their condition deteriorated within minutes to severe agitation. Such rapid change in behavior is frequently observed in cases of methamphetamine-induced intoxication and can be triggered by wait times or perceived threats [3, 15]. Clinical guidelines recommend verbal de-escalation and constant monitoring in an environment that is safe (without objects that could be used as weapons) and calm (to minimize over-stimulating triggers; [3, 15]).

If methamphetamine intoxication is not manageable, a benzodiazepine (e.g., lorazepam, diazepam) should be used as the medication of first choice for the acute treatment of agitation and distress [3, 15]. If the administration of benzodiazepines is not sufficient or is contra-indicated (e.g., in cases of mixed intoxication with respiratory depressants such as alcohol, opioids, or gabapentinoids), an antipsychotic (e.g., olanzapine, haloperidol) may be administered to reduce methamphetamine-induced agitation, especially when psychotic symptoms are present [3, 15]. Note that antipsychotics may potentiate methamphetamine-induced problems (in particular dysphoria, dyskinesia, seizures, and cerebrovascular events), which may complicate further management and reduce adherence to subsequent treatment.

In the present case, verbal de-escalation attempts by healthcare staff, including reassurance as well as offers of a calmer environment and anxiolytic medication, failed due to escalating paranoia and agitation. If cognitive impairment poses a substantial risk of significant harm to the individual or others (e.g., through traffic accidents, bodily harm), the least restrictive measures, up to physical restraint to allow for coercive medication, should be implemented to ensure the safety of patients, staff, and others [3]. However, physical restraint and coercive medication could not be implemented given the patient’s violent behavior in the absence of police and the lack of a controlled environment (e.g., seclusion room).

### Practical implications of ethical and legal considerations

This case report raises important ethical questions about the adequacy of risk management, particularly in balancing patient autonomy with the safety of the patient and others. Respecting patient autonomy, a fundamental ethical principle, is particularly challenging in emergencies such as methamphetamine intoxication, where impaired cognition and severe agitation may compromise the patient’s capacity for informed decision-making [1, 28].

Prioritizing safety can necessitate temporary limitations on autonomy, such as coercive measures, which must be carefully justified and be proportionate to the risk posed [1, 28].

From a medicolegal perspective, institutions bear significant responsibility in such high-risk cases. Clear documentation standards are critical, including detailed records of de-escalation attempts, the rationale for coercive measures, and post-incident reviews to ensure compliance with legal and ethical guidelines. Failure to maintain such standards may expose healthcare facilities to liability, particularly if adverse outcomes occur due to inadequate risk assessment or intervention protocols [3, 6].

The guideline-based management of methamphetamine intoxication necessitates the implementation of standard operating procedures by healthcare facilities in cooperation with local police. Healthcare teams and police forces must be regularly trained on these procedures as well as adequately staffed to effectively manage intoxicated and agitated individuals. Additionally, instructions must ensure that staff are trained not only in clinical management but also in understanding their legal obligations, including the need for transparent documentation to justify interventions and protect both patient rights and institutional accountability. Integration of technology, such as real-time decision-support tools for rapid tranquilization, has the potential to improve response times and protocol adherence.

## Conclusion

This case report describes an impulsive suicide after a downward spiral of life events, starting with suspected childhood ADHD, followed by chronic methamphetamine use, and culminating in socioeconomic adversity in adulthood. The patient’s history highlights systemic gaps in the effectiveness and continuity of addiction healthcare across treatment settings, particularly in managing methamphetamine use disorder and self-reported ADHD. Multiple detoxification and withdrawal treatments, including motivational and psychoeducational interventions, failed to establish a sustained therapeutic alliance, highlighting deficiencies in treatment integration and follow-up care. A comorbidity-sensitive approach is essential in addiction management; for example, suspected ADHD may initiate methamphetamine use as self-medication attempt, with escalating doses ultimately exacerbating symptoms resembling ADHD (inattention, hyperactivity, impulsivity) and potentially contributing to severe consequences, including psychosis and suicide.

Implementing integrated care models, such as collaborative care involving psychiatrists, addiction specialists, and primary care providers, could enhance treatment retention and address methamphetamine use holistically. While prescription stimulants can be clinically helpful for patients with ADHD, their misuse potential complicates clinical decision-making, especially in individuals with stimulant use disorder. Further research is urgently needed to develop evidence-based frameworks for weighing the risks and benefits of prescribing stimulants to adolescents and adults with comorbid ADHD and stimulant use disorder.

Finally, this case report underscores that the rapid escalation of agitation due to methamphetamine-induced intoxication and psychosis poses significant challenges for risk stratification and crisis management. The life-threatening risks to affected individuals and their surroundings necessitate the nationwide implementation of standard operating procedures with regular training and adequate staffing to manage agitated patients effectively and safely.

## Abbreviations

ADHD: Attention-deficit hyperactivity disorder
INCAS: International Naturalistic Cohort Study of ADHD and SUD
LSD: Lysergic acid diethylamide
MDMA: Methylenedioxymethamphetamine

## Declarations

No studies have been conducted on humans or animals for this case report. It is based on data from a postmortem forensic study [25] approved by the ethics committee at Dresden University of Technology (reference SR-EK-33012020). To protect the anonymity of the patient and staff, demographic and clinical details have been modified without altering any facts relevant to the discussion.
